# HOW MEDICAL OBJECTS, MEAL PREP ASSISTANCE, AND RELATIONAL CULTURE IMPACT THE MEANING OF “HOME” FOR HOME CARE CLIENTS

**DOI:** 10.1093/geroni/igae098.2949

**Published:** 2024-12-31

**Authors:** Lauren Spring, Stephanie Mason, Kaitlyn Kuryk, Janice Keefe, Kelly O’Neil

**Affiliations:** Mount Saint Vincent University, Halifax, Nova Scotia, Canada; Brock University, St. Catharines, Ontario, Canada; University of Manitoba, Winnipeg, Manitoba, Canada; Mount Saint Vincent University, Halifax, Nova Scotia, Canada; Mount Saint Vincent University, Halifax, Nova Scotia, Canada

## Abstract

Home care policies, programming, and services in Canada are designed to support older adult clients to age well in their homes and communities. Because the medical model dominates programming and policy decisions about home care services, the delivery of home care services is rarely viewed as relational and intra-active, in which interdependent relationships and interactions occur between clients and care workers. This paper applies a relational lens that incorporates a symbolic interactionist perspective to analyze qualitative data from a series of three interviews over an 18-month period (2019-2021) with 12 older adult home care clients and 10 of their paid caregivers. The overall study explored care pathways for older adult home care clients who received publicly-funded home care services in Manitoba and Nova Scotia. Our analysis revealed that this exchange of affect notably materialized through meal preparation and medical devices in the client’s home. The inter-/intra-actions between clients and care workers inscribed food-related practices and medical objects with symbolic values/meanings of home that were often unique and surprising. Better awareness of the relational cultures of meal preparation and medical devices to meanings of home and how these meanings for clients are sometimes in tension with those held by caregivers, can benefit clients and enrich home care policies and service delivery

